# Mitochondria-targeted antioxidant effects of S(-) and R(+) pramipexole

**DOI:** 10.1186/1471-2210-10-2

**Published:** 2010-02-05

**Authors:** Giulia Ferrari-Toninelli, Giuseppina Maccarinelli, Daniela Uberti, Erich Buerger, Maurizio Memo

**Affiliations:** 1 Department of Biomedical Sciences and Biotechnologies and National Institute of Neuroscience, University of Brescia, Brescia, Italy; 2 Department of Central Nervous System Research, Boehringer-Ingelheim Pharma, Biberach an der Riss, Germany

## Abstract

**Background:**

Pramipexole exists as two isomers. The S(-) enantiomer is a potent D_3_/D_2 _receptor agonist and is extensively used in the management of PD. In contrast, the R(+) enantiomer is virtually devoid of any of the DA agonist effects. Very limited studies are available to characterize the pharmacological spectrum of the R(+) enantiomer of pramipexole.

**Results:**

Using differentiated SH-SY5Y neuroblastoma cells as an experimental model, here we show that S(-) and R(+) pramipexole are endowed with equipotent efficacy in preventing cell death induced by H_2_O_2 _and inhibiting mitochondrial reactive oxygen species generation. Both pramipexole enantiomers prevented mitochondrial ROS generation with a potency about ten times higher then that elicited for neuroprotection.

**Conclusions:**

These results support the concept of both S(-) and R(+) pramipexole enantiomers as mitochondria-targeted antioxidants and suggest that the antioxidant, neuroprotective activity of these drugs is independent of both the chiral 6-propylamino group in the pramipexole molecule and the DA receptor stimulation.

## Background

Parkinson's disease (PD) is the most common neurodegenerative movement disorder. The primary cause of the disorder is the progressive loss of the pigmented dopaminergic neurons in the substantia nigra *pars compacta *(SNpc) accompanied by the appearance of intracytoplasmic inclusions known as Lewy bodies [1-3].

To date, the etiopathogenesis of nigral dopaminergic neuron loss in PD is unknown. However, the presence of ongoing oxidative stress as the result of compromised antioxidant defence mechanisms and generation of radical oxygen species (ROS) in the SNpc of the parkinsonian brain are considered to be important Pathogenic mechanisms [3,4]. ROS can react with cellular macromolecules through oxidation and cause the cells to undergo dysfunction and eventually lead to necrosis or apoptosis. The control of the redox environment of the cell provides an additional regulation in the signal transduction pathways which are redox sensitive. Therefore, an effective anti-parkinsonian therapy should not only alleviate the disease associated symptoms, but should also stop the progressive dopaminergic cell death in the SN.

Modification of the rate of PD progression is currently a highly debated topic. Increased oxidative stress is indeed thought to be involved in the nigral cell death which is a well established peculiar neuropathological feature of PD. These mechanisms have been proven *in vitro *and in animal models, but their relevance in humans remains speculative [5,6]. However, several dopamine (DA) agonists of the DA D2-receptor family (including D_2 _and D_3 _subtypes) have recently been shown to possess neuroprotective properties in different *in vitro *and *in vivo *experimental PD models [7,8]. At cellular level, independent groups have demonstrated decreased free radical production and an amelioration of DA neuronal loss following DA agonist treatment [9-17]. Interestingly, not all the neuroprotective effects were clearly mediated by DA-receptor stimulation.

Pramipexole (2-amino-4,5,6,7-tetrahydro-6-propylaminobenzathiazole) is a non-ergot DA agonist that has been successfully applied to the treatment of Parkinson's disease. Pramipexole exhibits a high affinity for the D_2 _and D_3 _DA receptor subtypes but little or no affinity for the D1 receptor family. The neuroprotective effects elicited by this drug have directly and/or indirectly been associated with antioxidant effects, mitochondrial stabilization or induction of the antiapoptotic Bcl-2 family [18-21]. In particular, Le et al., [18] reported that pramipexole protected DAergic MES 23.5 cell line against DA, 6-OH-DA and hydrogen peroxide (H_2_O_2_)-induced cytotoxicity possibly through antioxidant effects, and such neuroprotection was independent from DA receptor stimulation not being prevented by selective D_2 _or D_3 _antagonists. Similar results were obtained by Fujta et al., [20] and Uberti et al [22], who demonstrated that pramipexole inhibited generation of H_2_O_2_-induced reactive oxygen species in PC12 cells and SH-SY5Y neuroblastoma cells, respectively, in a DA receptor independent way. Recent data also demonstrated neuroprotection by pramipexole against β-amyloid ROS generation and toxicity [19,22].

Pramipexole exists as two isomers. The S(-) enantiomer is a potent D_2_/D_3_receptor agonist and is extensively used in the management of PD. In contrast, the R(+) enantiomer is virtually devoid of any of the DA agonist effects. Very limited studies are available to characterize the pharmacological spectrum of the R(+) enantiomer of pramipexole [19,22-27].

Here we show that S(-) and R(+) pramipexole are endowed with equipotent efficacy in preventing cell death induced by H_2_O_2 _and act as mitochondria-targeted antioxidants.

## Results

### Neuroprotection against H_2_O_2_

**Figure 1:**
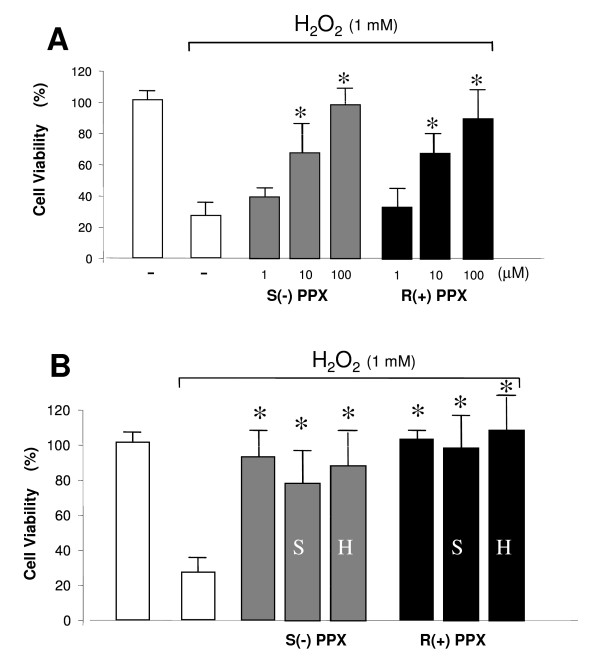
**Neuroprotective effects of pramipexole (PPX) enatiomers against H_2_O_2_-induced cell death**. A) Differentiated SH-SY5Y cells were exposed to different concentrations of S(-) PPX (gray bars) and R(+) PPX (black bars) for 1 h before being exposed to 1 mM H_2_O_2 _for 10 min. Cell viability was evaluated 24 h after by MTT assay B) Cells were exposed to 50 μM μM S(-) PPX or R(+) PPX in the presence or absence of 10 μM haloperidol (H) or 10 μM (-) sulpiride (S).  Data represent means ± SEM of at least three different experiments and are from three separate cell preparations. *, p < 0.01 vs H_2_O_2 _alone values.

SH-SY5Y neuroblastoma cell lines were differentiated with 10 μM all-trans retinoic acid for 1 week to acquire a neuronal phenotype. Cells were then challenged with 1 mM H_2_O_2 _for 5 min then cells returned to fresh medium for additional 24 h. H_2_O_2 _caused a reduction in cell viability of about 70% in comparison with untreated control cells (figure 1). As shown in figure 1A, treatment of the cells with increasing concentrations of S(-) or R(+) pramipexole dose-dependently prevented the viability impairment induced by H_2_O_2_. The tested drugs showed equipotent efficacy with calculated IC_50 _values of 8.8 ± 0.9 μM and 9.2 ± 0.6 μM for S(-) and R(+) pramipexole enantiomer, respectively. The neuroprotective effects of both pramipexole enantiomers were not prevented by preincubation of the cells with 10 μM phenoxybenzamine (data not shown), 10 μM haloperidol or 10 μM (-) sulpiride (Figure 1B). Haloperidol and sulpiride treatments did not induce cell viability modifications (data not shown).

### Inhibition of mitochondrial ROS generation

**Figure 2:**
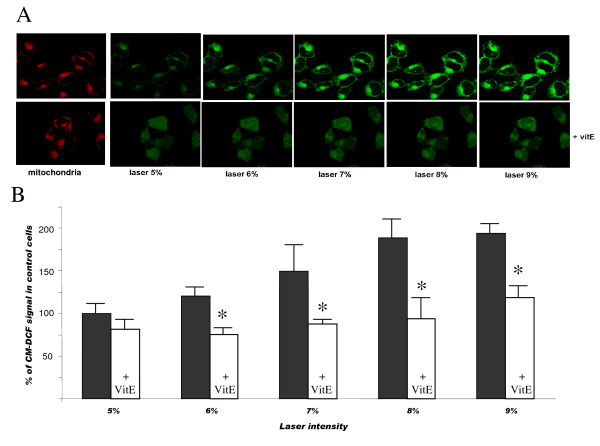
**Detection of mitochondrial ROS generation**. A) Upper panel. Representative pictures from cells exposed to increasing laser intensity, as indicated. CM-DCF fluorescence intensity (green) was selectively recorded in mitochondria (red). Lower panel. Cells were preincubated with Vitamin E (2 ng/100 μl) for 30 min before the laser excitation (white bars in panel B). Fluorescence emission intensity was calculated as average grey level value per pixel and corrected for background. Bars in panel B represent the means ± SEM of at least three different experiments and are from three separate cell preparations. *, p < 0.01 vs the corresponding control values.

The effects of pramipexole and its R(+) enantiomer have been studied in an experimental model of mitochondrial ROS generation by video-rate confocal microscopy in living neuronal cells. This model allows detection of ROS levels specifically generated in mitochondria and is based on the CM-DCF formation after laser light stimulation [28-30]. Figure 2A, upper panel, shows the results from a representative experiment. Mitochondrial ROS generation was evaluated in cells after increasing exposure to laser at different intensity. In a parallel experiment (lower panels), cells were preincubated with Vitamin E (2 μl/100 ml) for 30 min before the laser excitation. Fluorescence emission intensity was calculated as average grey level value per pixel and corrected for background. Data are reported in the graph reported in Figure 2B. The results clearly show that Vitamin E prevented laser-induced ROS generation in mitochondria of differentiated SH-SY5Y neuronal cells.

**Figure 3:**
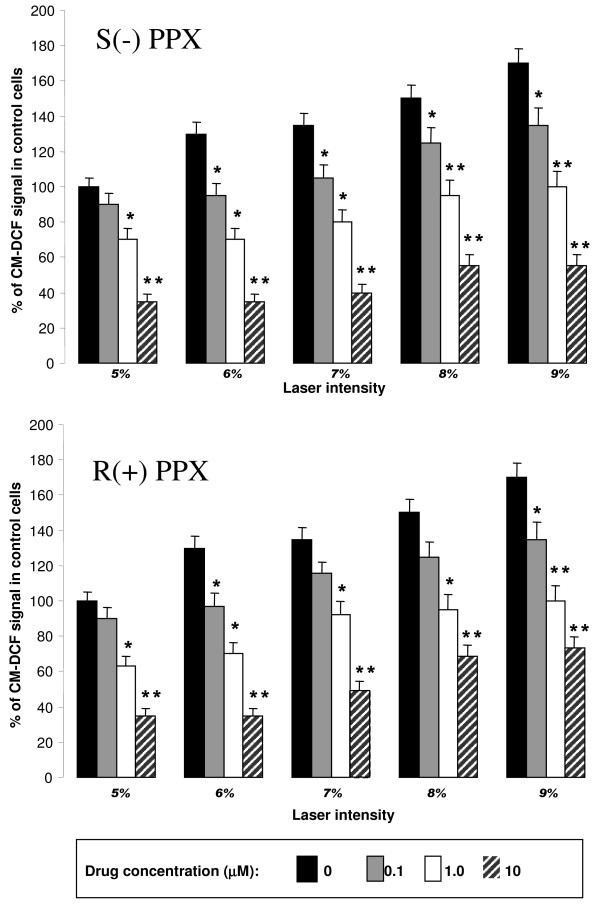
**Inhibition of mitochondrial ROS generation by pramipexole (PPX) enantiomers**. Cells were exposed to different concentrations, as indicated in the bottom, of S(-) PPX (upper panel) and R(+) PPX (lower panel) for 1 h before being exposed to laser. Mitochondrial ROS generation was evaluated as in figure 2. Data represent means ± SEM of at least three different experiments and are from three separate cell preparations. *, p < 0.05 and **, p < 0.001 vs the corresponding control values (black bars).

**Figure 4:**
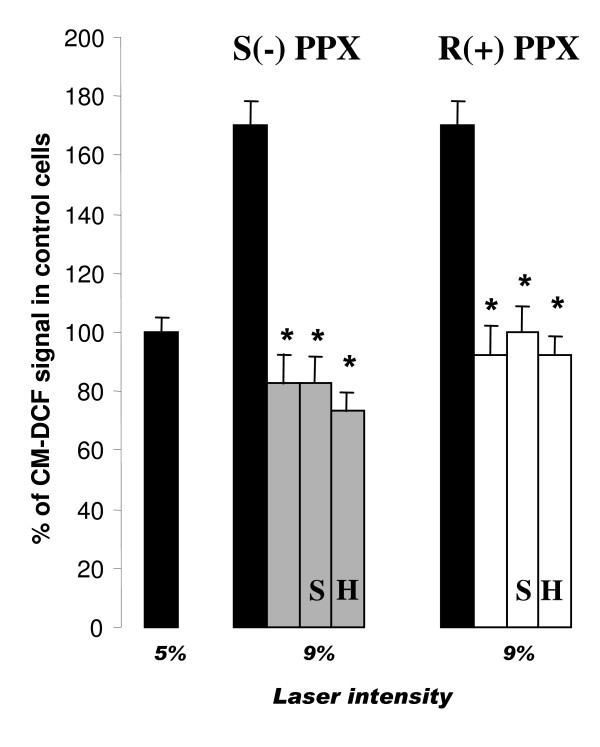
**Lack of effect of DA receptor antagonists on the inhibition of mitochondrial ROS generation induced by pramipexole (PPX) enantiomers**. Cells were exposed to 10 μM S(-) PPX (gray bars) and R(+) PPX (white bars) for 1 h in the absence or presence of 10 μM haloperidol (H) or 10 μM (-) sulpiride (S) before being exposed to laser. Mitochondrial ROS generation was evaluated as in figure 2. Data represent means ± SEM of at least three different experiments and are from three separate cell preparations. *, p < 0.01 vs the corresponding control values (black bars).

Using the same experimental paradigm, we tested the effects of different concentrations of S(-) and R(+) pramipexole in laser-induced mitochondria ROS generation. As shown in figure 3, both S(-) and R(+) pramipexole dose-dependently prevented laser-induced ROS generation in mitochondria of differentiated SH-SY5Y neuronal cells. When calculated as inhibition of ROS generation after 9% laser intensity, both drugs showed similar potency with IC_50 _values of 0.91 ± 0.14 μM and 0.85 ± 0.21 μM for S(-) and R(+) pramipexole enantiomer, respectively. The prevention of mitochondrial ROS generation induced by both pramipexole enantiomers was not affected by preincubation of the cells with 10 μM phenoxybenzamine (data not shown), 10 μM haloperidol or 10 μM (-) sulpiride (Figure 4). Haloperidol and sulpiride treatments did not modify laser-induced increase in ROS production (data not shown).

## Discussion

Pramipexole exists as two stereoisomers. The S(-) enantiomer is a potent D_2_/D_3 _receptor agonist and is extensively used in the management of PD. In contrast, the R(+) enantiomer is virtually devoid of any of the DA agonist effects. A growing number of experimental data indicate an antioxidant property of pramipexole enantiomers, evidenced by equal antioxidant efficacy toward H_2_O_2 _and nitric oxide [24] and equipotent efficacy in preventing viability impairment induced by H_2_O_2 _and mithocondrial ROS generation (present results). We found S(-) and R(+) pramipexole enantiomers relatively weak H_2_O_2 _scavengers, with apparent IC_50 _values in the low micromolar range. Our data are consistent with previous data showing neuroprotection elicited by the S(-) and R(+) enantiomers against different neurotoxic agents [9,22-27]. In our study, S(-) and R(+) pramipexole enantiomer showed equipotent efficacy suggesting that the neuroprotective effects against H_2_O_2 _in differentiated SH-SY5Y neuroblastoma cells were DA receptor independent.

Although this study did not examine the precise site of action of pramipexole, several finding implicate the permeability transition pore (PTP) as a possible target of this drug [19,24,25]. Apart from binding to DA receptors, pramipexole has in fact been shown to enter and accumulate in mitochondria driven by the mitochondrial membrane potential [24]. Targeting to mitochondria has also been recently demonstrated by patch clamp studies showing inhibition of PTP by pramipexole [25]. PTP inhibition by pramipexole was further supported by experimental data obtained in functional intact mitochondria showing that this drug abolished Ca^++^-triggered swelling [25]. By video-rate confocal microscopy in living neuronal cells, we found that both S(-) and R(+) pramipexole enantiomers prevented laser-induced ROS generation in mitochondria of differentiated SH-SY5Y neuronal cells. Interestingly, both pramipexole enantiomers prevented mitochondrial ROS generation with a potency about ten times higher then that elicited for neuroprotection.

The apparent discrepancy between the different potencies of pramipexole enantiomers in preventing mitochondrial ROS generation (about 0.9 μM) and inhibiting H_2_O_2_-triggered viability impairment (about 8 μM) may be related to the different experimental models. In fact, H_2_O_2 _itself is not a radical but reacts with iron to form hydroxyl radicals, the most reactive oxygen species. Thus, in our experimental paradigm, ROS are generated both intra- and extracellularly and causes apoptosis by the induction of several intracellular converging pathways involving lipid peroxidation, protein oxidation and DNA damage. We hypothesize that accumulation of promipexole enantiomers in the mitochondria [23,24] may limit their scavenger properties to specific subcellular compartments. Although accumulation of pramipexole into mitochondria has not been definitely established, the high potency of these drugs in preventing mitochondrial ROS generation strongly suggest the mitochondria as their primary site of action.

## Conclusions

These results support the concept of both S(-) and R(+) pramipexole enantiomers as mitochondria-targeted antioxidants and suggest that the antioxidant, neuroprotective activity of these drugs is independent of both the chiral 6-propylamino group in the pramipexole molecule and the DA receptor stimulation.

## Methods

### Cell culture

The human neuroblastoma cell line SH-SY5Y was routinely cultured in Ham's F12 and Dulbecco modified Eagle's medium (DMEM) in a ratio of 1:1, supplemented with 10% (v/v) foetal calf serum, 2 mM glutamine, 50 μg/ml penicillin, and 100 μg/ml streptomycin and kept at 37°C in humidified 5% CO_2_/95% atmosphere. For differentiation, cultured cells were treated for one week with 10 μM all trans retinoic acid. To obtain reproducible results, SH-SY5Y cells ranging from passage 18 to passage 25 were used for all the experiments.

### Drug treatment

S(-) and R(+) pramipexole were dissolved in water and added to the culture media 1 h before H_2_O_2 _pulse or laser light stimulation. IC_50 _values for S(-) and R(+) pramipexole enantiomer were calculated using at least 5 data points. Haloperidol and sulpiride were added to the culture media 1 h before pramipexole. Vitamin E (2 ng/100 μl) was added to the culture media 30 min before the laser excitation. All drugs were from Sigma. S(-) and R(+) pramipexole were kindly supplied by Boehringer Ingelheim GmbH, Germany.

### Evaluation of cell viability

Cell viability was measured by quantitative colorimetric assay with MTT (3-(4,5-dimethylthiazol-2-yl)-2,5-diphenyltetrazolium bromide), showing the mitochondrial activity of living cells. Differentiated SH-SY5Y neuronal cells in 96-well plates were challenged with H_2_O_2 _for 5 min, then 500 μg/ml MTT was added in each well and cells were incubated at 37°C for additional 3 h. MTT was removed, and cells were lysed with dimethyl sulfoxide. The absorbance at 595 nm was measured using a Bio-Rad 3350 microplate reader. Control cells were treated in the same way without H_2_O_2_, and the values of different absorbances were expressed as a percentage of control. All the drugs and reagents concentrations are to be considered as final concentrations.

### Reactive oxygen species detection

SH-SY5Y neuroblastoma cells were first differentiated in neuronal-like phenotype by treatment with retinoic acid for 7 days; then, according to Koopman et al. [28], cells were loaded with 5-choloromethyl-2',7'-dichlorodihydrofluorescein (CM-H_2_DCF) and its oxidative conversion into CM-DCF was monitored by video-rate confocal microscopy and real-time image averaging after increasing laser intensities. For mitochondria localization, cells were also loaded with Mitotracker Deep Red. Cells were then excited at increasing laser intensity and CM-DCF fluorescence intensity was selectively recorded in mitochondria. Fluorescence emission intensity was calculated as average grey level value per pixel and corrected for background.

### Statistical evaluation

Results were given as mean ± standard error mean values. Statistical significance of differences was determined by one way ANOVA, followed by Bonferroni's multiple comparison test as post-hoc analysis. A probability of less than 0.05 was considered as a significant difference.

## Authors' contributions

GFT, GM and DU conducted and managed the study and analyzed the imaging data, MM drafted the manuscript. EB and MM participated in the design and coordination of the project. All authors cooperatively designed the project and discussed data interpretation. All authors participated in critical editing of the manuscript.
